# Insights into Paramyxovirus Nucleocapsids from Diverse Assemblies

**DOI:** 10.3390/v13122479

**Published:** 2021-12-10

**Authors:** Tianhao Li, Qing-Tao Shen

**Affiliations:** 1 iHuman Institute, School of Life Science and Technology, ShanghaiTech University, Shanghai 201210, China; lith@shanghaitech.edu.cn; 2 Laboratory for Marine Biology and Biotechnology, Qingdao National Laboratory for Marine Science and Technology, Qingdao 266237, China

**Keywords:** *Paramyxoviridae*, nucleoprotein, nucleocapsid, clam-shaped structure, double-headed nucleocapsids

## Abstract

All paramyxoviruses, which include the mumps virus, measles virus, Nipah virus, Newcastle disease virus, and Sendai virus, have non-segmented single-stranded negative-sense RNA genomes. These RNA genomes are enwrapped throughout the viral life cycle by nucleoproteins, forming helical nucleocapsids. In addition to these helical structures, recombinant paramyxovirus nucleocapsids may occur in other assembly forms such as rings, clam-shaped structures, and double-headed nucleocapsids; the latter two are composed of two single-stranded helices packed in a back-to-back pattern. In all of these assemblies, the neighboring nucleoprotein protomers adopt the same domain-swapping mode via the N-terminal arm, C-terminal arm, and recently disclosed N-hole. An intrinsically disordered region in the C-terminal domain of the nucleoproteins, called the N-tail, plays an unexpected role in regulating the transition among the different assembly forms that occurs with other viral proteins, especially phosphoprotein. These structures, together with the helical nucleocapsids, significantly enrich the structural diversity of the paramyxovirus nucleocapsids and help explain the functions of these diverse assemblies, including RNA genome protection, transcription, and replication, as well as encapsulation.

## 1. Introduction

**Figure 1:**
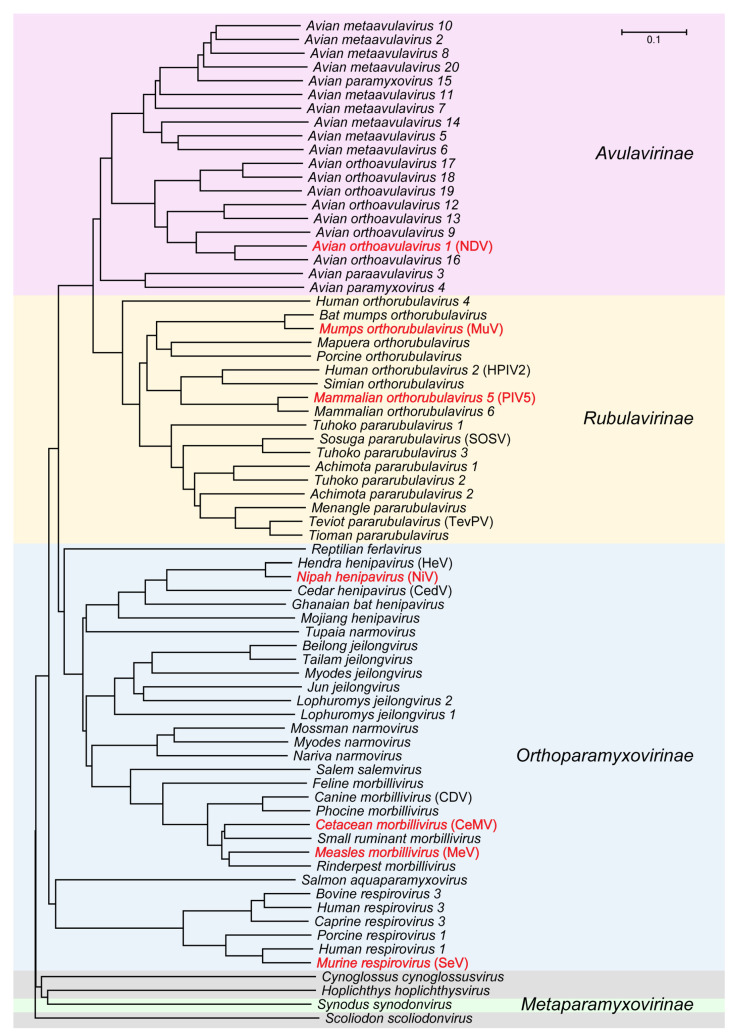
Phylogenetic tree based on the nucleoprotein sequences in family of *Paramyxoviridae*. *Cynoglossus cynoglossusvirus*, *Hoplichthys hoplichthysvirus*, and *Scoliodon scoliodonvirus* are not classified into any subfamily yet. Nucleoproteins with available protein structures within four subfamilies are colored in red. Phylogenetic tree is constructed via neighbor-joining method with bootstrap values determined by 1000 replicates in MEGAX [3].

*Paramyxoviridae* is a family of single-stranded negative-sense RNA viruses in the order of *Mononegavirales* [1]. This family has four subfamilies, including *Avulavirinae*, *Rubulavirinae*, *Metaparamyxovirinae*, and *Orthoparamyxovirinae*, and consists of 78 species [1]. Most paramyxoviruses are either human viruses, such as the measles (MeV), parainfluenza (PiV), and mumps viruses (MuV), or animal-derived pathogens, such as the Newcastle disease (NDV) and Sendai viruses (SeV) (Figure 1). Although vaccines are available for some paramyxoviruses, such as MeV and MuV, related diseases reemerge in many regions of the world and even occur in people with a history of vaccination. These cases can cause serious health problems and even death [2].

Paramyxoviruses are enveloped spherical virions that vary from about 150 to 300 nm in size. Their genomes are usually 15–19 kilobases in length [4]. Similar to other mononegaviruses, paramyxovirus genomes are non-segmented and contain six to ten viral genes. Paramyxoviruses take advantage of a single promoter mode by having their genes arranged in the following order: nucleoprotein (N)—phosphoprotein (P)—matrix (M)—fusion (F)—hemagglutinin (H/HN/G)—large (L), and their expression levels meet the optimal requirements for successful infection.

Nucleoproteins are expressed at the highest level among all viral proteins since they are required in large amounts to fully coat nearly every nucleotide of the paramyxovirus RNA genome. The nucleoproteins coil the RNA genomes into helical nucleoprotein–RNA complexes (nucleocapsids), which are critical for a variety of functions. Nucleocapsids play critical roles in the RNA genome protection against nuclease, RNA genome transcription and replication, as well as virion maturation [5,6]. Nucleocapsid assemblies of mononegaviruses have been comprehensively reviewed by Kolakofsky, Luo, and Lou et al. [5,7,8]. In this review, we focus on the recent progress on paramyxovirus nucleocapsids, especially related to their different high-order assembly forms. We also discuss the molecular mechanism of the diverse assemblies and the biological relevance.

## 2. Sequence and Structure Similarity

Most paramyxovirus nucleoprotein genes have nucleotides in a tight range of 1500–1700 bases, and the corresponding nucleoproteins possess about 520 amino acids at the protein level. A detailed sequence alignment shows that the sequence among the paramyxovirus nucleoproteins is quite conserved. SeV, PiV5, NDV, MeV, MuV, Nipah virus (NiV), and *Cetacean morbillivirus* (CeMV) have the highest sequence similarity of nucleoprotein at a level of 0.75. As the control, other viral proteins in these viruses, such as phosphoprotein and hemagglutinin protein, only have a sequence similarity of 0.48 and 0.44, respectively.

**Figure 2:**
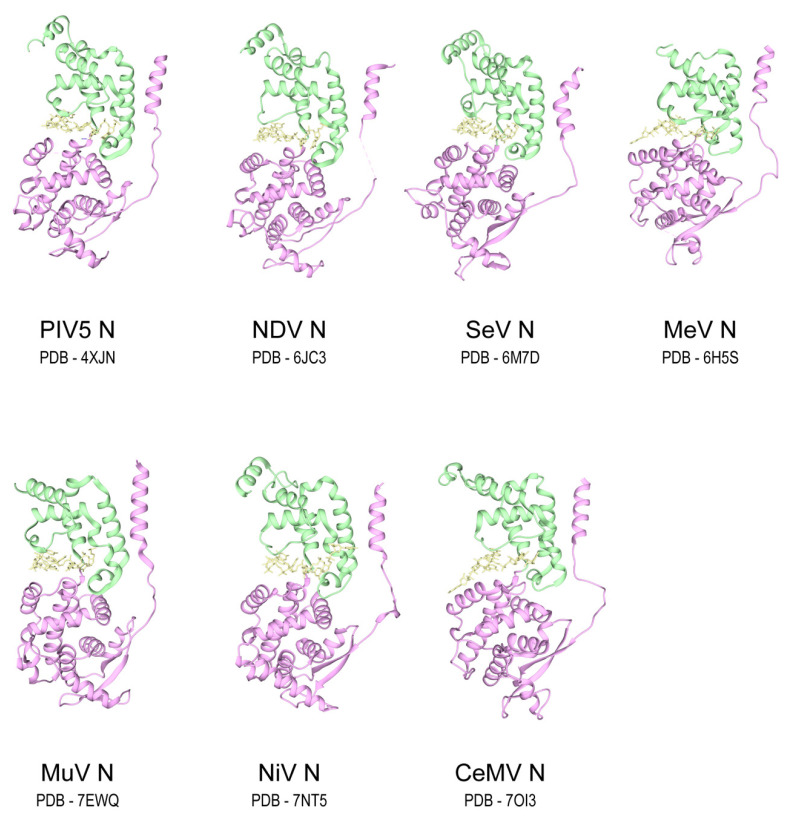
Nucleoprotein protomers of seven paramyxovirus nucleocapsids. NTD and CTD of nucleoproteins, and RNA hexamer are colored in pink, green, and yellow, respectively.

Among the 78 paramyxovirus nucleoproteins, 7 of them have been resolved in the context of their respective nucleocapsids at near-atomic resolutions via either X-ray crystallography or cryo-electron microscopy (cryo-EM) (Figure 2) [9,10,11,12,13,14,15]. Conforming well with the idea of high sequence similarity, these paramyxovirus nucleoproteins exhibit a high structural similarity with a root-mean-square-deviation (R.M.S.D) of less than 1.3 Å. All paramyxovirus nucleoproteins have the same domain organization, including the N-terminal domain (NTD) and the C-terminal domain (CTD). The NTD usually consists of about 250 residues and is more conserved than CTD. The core of the NTD is globular in shape and consists of nine helices. In the NTD, there is an extended N-terminal arm (N-arm) packed against the core of the NTD from the upstream protomer. The N-arm connects the core of the NTD via an extended loop (Loop_20–46_). The Loop_20–46_ is surprisingly inflexible in structure and has been found at high resolution in almost every well-resolved paramyxovirus nucleocapsid (Figure 2) [9,10,11,12,13,14,15].

The CTD of a paramyxovirus nucleoprotein has about 200 residues and also exists in a globular shape. Similar to the NTD, the CTD has a C-terminal arm (C-arm) that can bind to the core of the downstream CTD. Immediately following the C-arm, there is an intrinsically disordered C-terminal domain called the N-tail. The N-tail has poor sequence similarly among the paramyxovirus nucleoproteins and is quite flexible in structure. So far, no integral structural information has been resolved for the N-tail via cryo-EM, and the first 12 residues of the N-tail are resolved in the SeV nucleoprotein, which assembles into a loop and points outward from the helical nucleocapsids [10]. Nuclear magnetic resonance (NMR) spectroscopy provides more structural information of the intact nucleoprotein itself, along with the NTD of P at atomic resolutions [16,17]. NMR spectroscopy allows an atomic resolution description on flexible regions especially the N-tail in the highly dynamic N^0^P complex. Derived from these NMR results, the N-tails are also supposed to point outward in the nucleocapsids [18].

In paramyxovirus nucleoproteins, the NTD and CTD are tightly attached to each other, with a cleft in between. In the MeV nucleoprotein, the cleft is composed of several residues, such as T_183_, K_180_, R_194_, R_195_, Q_202_, Y_260_, A_267_, and R_354_ [12,19]. These charged residues are quite conserved among all paramyxovirus nucleoproteins and are essential to bind the negatively charged RNA nucleotides via electrostatic interactions [9,10,11,13,14,15]. Each nucleoprotein binds six nucleotides in a “three-bases-in, three-bases-out” pattern [20,21]. Interestingly, the genome sizes of paramyxoviruses are found to be the multiples of six (the “rule of six”), which is critical for genome transcription and replication. Almost all nucleotides of the RNA genome will be covered by the nucleoproteins by assembling into high-order structures, except that the last three nucleotides at the 3′ end of the nucleocapsid are largely exposed, possibly enabling access to the viral polymerase [19].

## 3. Ring-like Nucleocapsids

**Figure 3:**
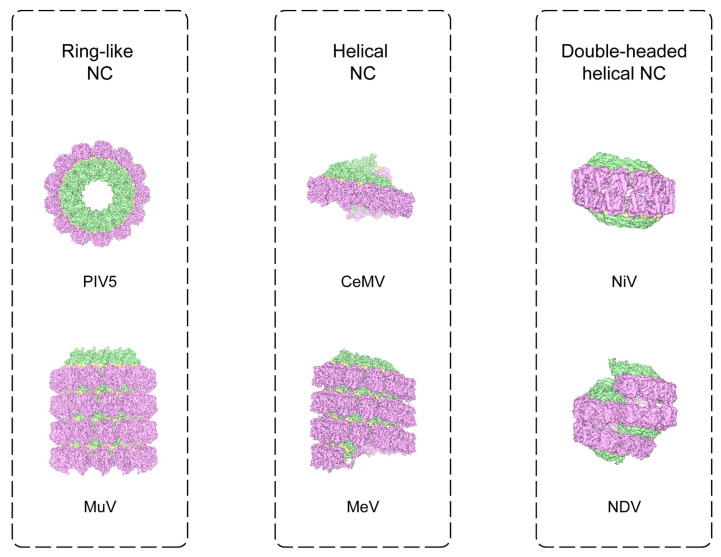
Different assembly forms of selected paramyxovirus nucleocapsids or nucleocapsid-like particles in vitro. Nucleocapsids of PIV5 and CeMV are shown in surface representation. Virus names and EM data bank accession numbers are as follows: MuV 30266; MeV 2867; NiV 12582; NDV 9793. NTD and CTD of nucleoproteins, and RNA polymers are colored in pink, green, and yellow, respectively.

Purified paramyxovirus nucleoproteins do stay in the high-order assemblies instead of a monomeric state. Early studies have shown that ring-like assemblies co-exist with the helical forms of the full-length purified paramyxovirus nucleoproteins via negative-stain EM [22]. Limited by the resolution, only the protomer number per ring could be clearly recognized. Reference-free alignment shows that the *Morbillivirus* MeV rings have 13 protomers, while the *Orthorubulavirus* simian virus type 5 (SV5) has 14 protomers (Table 1) [22]. In later research, X-ray crystallography unveils that PIV5 (formerly known as SV5) has the protomer number at 13 [13]. Freshly purified MuV nucleoproteins also exhibit ring-like assemblies (Figure 3) [9]. The cryo-EM analysis indicates that there are two kinds of MuV rings with 13 and 14 protomers, respectively. The variation in protomer numbers in the MuV ring-like structures indicates the structural plasticity that is inherent in paramyxovirus nucleoproteins [9].

The high-resolution ring-like nucleocapsids from PIV5, MuV, and other viruses are available after the removal of flexible regions [9,13]. The RNAs are always visible in the ring-like assemblies and purified nucleoproteins enwrap the RNAs from the cell host without specificity. Structural analysis provides direct evidence that the RNA is clamped in the cleft between the NTD and CTD. In paramyxovirus ring-like nucleocapsids, the RNAs face outward, which means they are easily accessed by the P, L, and other factors. Similar RNA orientation is observed in the nucleocapsids of the rabies virus and other mononegaviruses [23]. The only exception is the ring-like nucleocapsid of vesicular stomatitis virus (VSV) in the family of *Rhabdoviridae*, which has its RNAs facing inward [14]. This reversed topology might lead to differing results related to the VSV during genome transcription and replication. However, the occurrence of the VSV ring-like nucleocapsid requires more in vivo data and further verification to exclude the possible crystal packing artifacts.

After incubation at 4 °C for several weeks, residual impurities can partially cleave the flexible regions, especially the N-tail from the MuV nucleoproteins [9,24,25]. The aged MuV nucleoprotein shows several bands from about 45 to 55 kDa with the N-tail or C-arm removed in SDS-page gel. Surprisingly, this treatment turns the MuV ring-like assemblies into filaments. The cryo-EM analysis on the MuV nucleocapsids shows that one kind of filament is composed of stacked rings (Figure 3) [9]. The rings with 13 protomers are packed into the layers in a head-to-tail pattern, and the neighboring layers have a twist angle of 9.3° so that one protomer from the upper layer can slide into the gap between the two neighboring protomers in the lower layer [9]. This close contact between the neighboring layers forms a stable interface with positively charged α16 helices from the N_i_ and negatively charged Loop_20__–46_ from the upper N_i_′ [9]. Similar to the ring-like nucleocapsids, each MuV layer possesses limited (~78) RNA nucleotides. Furthermore, the RNAs in the MuV neighboring layers cannot connect to each other. Thus, it is impossible for the stacked rings to enwrap the non-segmented genome, and either the ring-like nucleocapsids or the stacked rings are not biologically relevant.

## 4. Helical Nucleocapsids

The helical nucleocapsids seem to be the optimal form for paramyxoviruses to enwrap single-stranded RNA genomes. Studies on isolated SeV nucleocapsids have unveiled long helical filaments under the negative-stain EM [26,27] and found that these helical filaments can coexist in different conformations with the respective helical pitches of 5.3, 6.8, and 37.5 nm [27]. Cryo-electron tomography (cryo-ET) on the intact NDV, SeV, and MeV virions also indicates the existence of several long nucleocapsid filaments [28,29,30,31]. Analyses of the relatively straight nucleocapsids within the MeV virion point to the helical assemblies with helical pitches varying from 7.3 to 8.3 nm [29,31]. Interestingly, the helical nucleocapsids in NDV and SeV remain associated with, and in close proximity to, the matrix lattice, which might facilitate the assembly of helical nucleocapsids [29,31]. The neighboring helical nucleocapsid filaments within a virion are connected with highly curved or even bent loops. These flexible structures provide plasticity for paramyxovirus nucleoproteins to pack the RNA genomes into the tiny virions but make it difficult to visualize the high-resolution reconstruction of the intact nucleocapsids.

The purified, full-length nucleoproteins from NDV and SeV can also assemble into helical filaments, and these filaments are usually quite curved. This is similar to the nucleocapsids in a virion [10,11]. The first high-resolution helical nucleocapsids were observed in the MeV at a resolution of 4.3 Å after the truncation of the flexible N-tail (Figure 3) [12]. Removal of the N-tail tightens the MeV helical nucleocapsids into long and straight filaments, which are more suitable for the helical analysis of cryo-EM. The helical twist and helical pitch of the MeV nucleocapsids are −29.2° left-handed and 5.0 nm [12], respectively. Recently, the helical structures of the SeV were resolved at the high resolutions of 2.9 and 3.9 Å [10]. The two different conformations of the SeV helical nucleocapsids that have a small variability in helical pitch and helical twist are sorted out during 3D classification [10]. In addition to the stacked-ring filaments, the aged MuV nucleoproteins can assemble into helical nucleocapsids [9]. Interestingly, the two conformations with distinct helical pitch and helical twist were revealed from the same filaments. These two assemblies are referred to as MuV NC_helix-dense_ and NC_helix-hyper_ [9]. The helical pitches of MuV NC_helix-dense_ and NC_helix-hyper_ are 5.3 and 4.6 nm, respectively, with distinct differences. The helical twists of MuV NC_helix-dense_ and NC_helix-hyper_ are quite close at −27.1° and −26.8°, respectively. The corresponding protomers per turn are about 13.2 and fall between the MuV 13-protomer and 14-protomer rings. The compatibility of the helical and ring-like assemblies indicates a similar assembly mechanism.

## 5. Assembly Mechanism

The ring-like and helical assemblies of paramyxovirus nucleocapsids oligomerize via the same domain-swapping mechanism. Specifically, the N-arm from N_i_ that lies between the two α12 helices from N_i_ and N_i–1_, which assemble into a bundle with three anti-parallel helices. The other side of the α12 helix from N_i_ is unoccupied and can interact with the N-arm and the α12 helix from N_i+1_ [9,10,11,12,15,19]. The N-arm is strictly required for the assembly of paramyxovirus nucleocapsids, and the removal of the N-arm will abolish the assembly of the helical or ring-like structures, as revealed in NDV [11]. In addition to the N-arm, the C-arm from N_i_ interacts with the α16 helix of N_i+1_ to capture the subsequent nucleoprotein. It is also involved in the assembly of nucleocapsids via the domain-swapping mechanism. The C-arm may not be as essential as the N-arm for the assembly of helical nucleocapsids. The truncation of the C-arm has been reported not to affect the assembly into the helical nucleocapsids in MuV and NDV [9,11].

In addition to the N-arm/C-arm domain-swapping, another new interface was observed in the SeV nucleocapsids with high resolutions [10]. In the SeV ring-like and helical nucleocapsids, the extended Loop_20–46_, together with Loop_92–102_ from the NTD and Loop_312–320_ from the CTD, form a closed, hole-like structure denoted as the N-hole. The paired structure from the upstream protomer, Loop_240–248_, pops out from the NTD of N_i–1_ with a size of 8 Å. Loop_240–248_ fits in well with the size of the N-hole and can be inserted into the N-hole from N_i_ via electrostatic interactions. The replacement of all negatively charged residues in Loop_240–248_ with alanine will abolish the electrostatic interactions with the N-hole and yield threading thin filaments. This verifies a specific role in the assembly of helical and ring-like nucleocapsids [10].

Similar N-hole structures also exist in NDV, PIV5, and MeV with well-resolved structures [10,11,14]. Detailed structural analyses indicate the occurrence of electrostatic interactions between the N-holes and the extended loops in these paramyxovirus nucleoproteins [10]. Thus, these conserved interfaces between the N-holes and the extended loops resemble a gate’s latch and bolt. These interfaces apparently function to tightly anchor the positions of the neighboring nucleoprotein protomers. Based on the sequence similarity, this new interface should be conserved among the family of *Paramyxoviridae*.

The RNA strands are always visible in the paramyxovirus nucleocapsids, and the depletion of RNA from the MuV nucleocapsids renders a more flexible filament [25]. These qualities suggest that RNA plays roles in the assembly of nucleocapsids. The addition of RNA can trigger the MeV and CeMV N^0^P fusion proteins to initiate self-assembly of nucleocapsid-like particles, and the assembly depends strongly on the RNA sequences [32,33]. In MeV, the genomic 5′ end and poly-adenine sequences help efficiently assemble the nucleoproteins into nucleocapsid-like particles, while other sequences, such as poly-uracil, cannot [32]. A structural comparison of the nucleoprotein protomer in the MeV nucleocapsid-like particles with N^0^P reveals a significant reorientation of the N-arm and C-arm as well as conformational change involving the two helices that form the edges of the RNA-binding groove [5]. The role of RNA in the nucleocapsid assembly seems highly related to the structural transition of nucleocapsids among different forms.

## 6. Double-Headed Nucleocapsids

In some cases, one SeV virion may contain one to six copies of the genome, which enlarges the virion size from 110 to 540 nm [30]. Two or three genomes per virion also occur in NDV and MeV [34,35]. The packaging of the multiple genomes does not affect the growth of MeV [35]. Each RNA genome of the polyploid paramyxoviruses will be packaged via the nucleoproteins into a helical nucleocapsid. It remains to be discovered exactly how the polyploid paramyxoviruses orchestrate their multiple nucleocapsids into one virion. Recently, a clam-shaped assembly was uncovered from purified NDV nucleoproteins. The cryo-EM reconstruction shows that the NDV clam-shaped assembly is composed of two single-turn spirals packed in a back-to-back pattern (Figure 3) [11]. Each single-turn spiral has an individual RNA strand, and an obvious seam separates the two RNA strands from the single-turn spirals. The protomers further away from the seam are better visualized than those closer to the seam.

In the NDV clam, the assembly of the single-turn spiral follows the same mechanism as the helical nucleocapsids including the swapped N-arm, C-arm, and N-hole. Two single-turn spirals form a new interface to hold these two opposite spirals together. The interface comes from Loop_114–120_ of the vertically adjacent protomers in the clam-shaped assembly. A distance analysis of the residues in Loop_114–120_ suggested that hydrogen bonds may exist between the two pairs of R_117_ and G_119_ residues. The replacement of Loop_114–120_ with all alanine will only yield single-turn spirals. Loop_114–120_ appears to be involved in the oligomerization of the clam-shaped assembly but not in the helical assembly of the single-turn spirals [11].

Similar clam-shaped structures have been reported in purified SeV and NiV nucleoproteins (Figure 3, Table 1) [10,36]. Both the SeV and NiV assemblies adopt the same back-to-back pattern as the NDV clam to connect the two single-turn spirals together. Compared with the relatively loose interface in the NDV clam, both the SeV and NiV clams adopt a tightly crisscrossed pattern. This tighter and more engaged pattern clearly impacts the capacity for lateral sliding between the two opposite single-turn spirals [10]. Similar to the NDV clam, the single mutation of F_118_ into alanine on the SeV nucleoproteins can also abolish the assembly of the SeV clam [10]. The sequence alignment on the nucleoproteins shows a highly conserved “FxxK” motif ranging from 118 to 121 on NDV, SeV, NiV, and MeV. This verifies the popularity of clam-shaped structures in the family of *Paramyxoviridae* [11]. Not limited to the paramyxovirus, the two ring-like nucleocapsids of the rabies virus also take a back-to-back pattern in the unit cell of crystal, which uses two opposite Loop_121–130_ to hold two rings together [23]. Obviously, the occurrence of the clam-shaped structures in mononegaviruses needs direct cryo-EM evidence from more recombinant nucleoproteins.

Only 12–14 nucleoprotein protomers can be clearly recognized in the cryo-EM map for each single-turn spiral of the NDV clam [11], which limits its biological relevance. Interestingly, in each single-turn spiral of the NDV clam, the 14th protomer lies above the 1st protomer and starts growing into the 2nd round of helix [11]. The NDV clam has the potential to grow further into a double-headed filament following this helical trajectory. During the purification of NDV nucleoproteins, there are double-headed nucleocapsid filaments accompanied by clam-shaped particles. In purified SeV nucleoproteins, double-headed nucleocapsid filaments are more dominant [10]. The clam joints in the SeV double-headed nucleocapsids were utilized as the side views during the 3D reconstruction of the SeV clam [10]. The helical pitch and helical twist in the SeV clam are 5.6 nm and −27.1°, respectively, similar to the values of the SeV double-headed nucleocapsids [10]. This evidence supports the structural compatibility of the clam-shaped assemblies and the double-headed nucleocapsids.

Derived from clam-shaped assemblies, double-headed nucleocapsids contain two long RNA strands, which are also separated by a seam in the clam joint. Interestingly, NDV, MeV, and SeV have multiple copies of genomes in one virion, and these copies of genomes are more readily available in even numbers. Considering that both NDV and SeV are capable of assembling into double-headed nucleocapsids, these double-headed nucleocapsids may efficiently pack polyploid genomes into one virion in SeV and NDV [30,34]. Whether MeV can assemble into double-headed nucleocapsids or how MeV orchestrates its genome requires further investigation.

Another possible role for the double-headed nucleocapsids is to provide protection against nuclease in NDV. Compared with the NDV wild-type double-headed nucleocapsids, the single-headed nucleocapsids from the Loop_114–120_ replacement mutation are more sensitive to nuclease and protease [11]. It is speculated that the two spirals in double-headed nucleocapsids can cap each other and protect the susceptible 5’ ends from possible degradation [11].

## 7. Structural Transitions

Different assembly forms in the family of *Paramyxoviridae*, including rings, stacked-rings, clam-shaped structures, and the derived double-headed nucleocapsids, have been observed at high resolutions [9,10,11,12,13,15,36]. Although the N-tails are usually poorly resolved, in part due to their intrinsic flexibility, they, together with other viral proteins (especially P), are capable of regulating the structural transition among different assembly forms.

During RNA synthesis, the SeV P–L RNA polymerase complex binds to the viral nucleocapsid template through an interaction of the CTD of P with the N-tail of nucleocapsids [37]. Binding of P may initiate the uncoiling of nucleocapsids, as has been observed with MuV, to facilitate the release of the genomic RNA 3′ end from the RNA-binding groove [25]. The newly synthesized RNA will become encapsidated via interacting with a monomeric nucleoprotein, which is also regulated by P. P is reported to utilize its 48 N-terminal amino acids to bind to the newborn nucleoproteins, which keeps the nucleoproteins in the monomeric state, or N^0^P [38,39,40,41,42]. In the crystal structure of MeV N^0^P, the interaction of P with a nucleoprotein is mainly hydrophobic, and this interface inhibits the association of the adjacent protomers with the growing nucleocapsids by steric hindrance [41]. Nucleoprotein and P have plenty of intrinsically disordered regions, and their mixture is reported to form liquid-like phase-separated micrometer-scale inclusion bodies in vivo [43] and micrometer-scale droplets in vitro [44]. The minimal elements of the droplets are determined as P_MD_, P_LOOP_, P_XD_, and the integral N. The RNA can colocalize to the MeV droplets, which triggers the assembly of nucleocapsids [44]. Apparently, P regulates the structural transition between the monomeric state and the oligomeric state of nucleoproteins and is also involved in the uncoiling of the helical nucleocapsids. How P orchestrates both activities during genome transcription/replication is worthy of further investigation.

The N-tails are involved in the transition from rings to helical nucleocapsids. Purified full-length MuV nucleoproteins always exist in ring-like assemblies [9,45]. The N-tail can be removed via either truncation mutation or protease cleavage. Interestingly, the absence of the N-tail turns the rings into three different kinds of nucleocapsid filaments, including stacked ring, NC_helix-dense_, and NC_helix-hyper_ [9]. Obviously, the N-tail plays a negative role in switching from the rings to helical nucleocapsid filaments. Under native environments, the CTD of P might relocate the N-tail to a new position for the assembly of the helical nucleocapsids.

The N-tail is not limited to the transition from the rings to helical filaments; it also plays key roles in regulating the curvature of the helical nucleocapsids. Full-length SeV helical nucleocapsids are usually highly curved, and the persistence length of curved SeV helical nucleocapsids is about 288 nm [10]. When the N-tail is removed from the full-length SeV nucleoproteins, the yielded nucleocapsids are much straighter, with a typical persistence length of about 877 nm [10]. Such straight nucleocapsids are homogenous in their helical rises and twists, making them perfect for a high-resolution cryo-EM analysis. This explains why most nucleocapsids are resolved with their N-tails cleaved or truncated. 

The C-arms are also reported to play a role in regulating the MuV assembly from NC_helix-dense_ to NC_helix-hyper_ [9]. More specifically, when the C-arm is removed via residual impurities after a long-time incubation, the MuV nucleocapsids will transit from a dense state to a hyper-dense state. This means that the helical pitch drops from 5.3 to 4.6 nm [9]. The location of the C-arm may be highly relevant to the assembly of the MuV nucleoproteins into either dense or hyperdense MuV nucleocapsids [9].

## 8. Perspective on Diverse Assemblies

Nucleocapsids from different paramyxoviruses have different assembly forms, and even nucleocapsids from the same paramyxovirus may also exhibit diverse assemblies. In one SeV nucleocapsid, clam-shaped joints and straight/condensed filaments coexist with loosed or even uncoiled filaments [10]. Compared with straight fragments, the curved filaments may have their N-tails exposed to the CTD of P and can therefore regulate gene transcription and replication. Clam-shaped structures will protect the integrity of the RNA genome and orchestrate multiple RNA genomes in one virion. Thus, the diverse assemblies in double-headed SeV nucleocapsids represent distinct functions. This idea might be applied to other paramyxoviruses.

The encapsulation of paramyxovirus nucleocapsids in virions offers the opportunity to closely examine the native assemblies and the in vitro observations. Cryo-ET and tomography averaging on intact virions will be useful to provide evidence for the structural basis of the paramyxovirus nucleocapsids in a compact state. During genome transcription and replication in a cell host, other viral proteins such as P and L, will become involved in regulating the assembly of paramyxovirus nucleocapsids. The direct examination of the N-P-L super complex will inevitably be limited due to complicated environments. An in vitro reconstitution of the N-P-L complex would be a better choice for a high-resolution structural analysis. The MuV rings may be a good scaffold to recruit the P-L complex, and a fixed number of protomers will make the high-resolution cryo-EM analysis feasible.

In summary, the recent structural studies have found several peculiar assembly forms, especially the clam-shaped structures and derived double-headed nucleocapsids. These forms have not been reported previously in paramyxoviruses or other mononegaviruses. Further work aiming at correlating the various observed assemblies of nucleocapsids with their functions is needed.

## Figures and Tables

**Table 1:** Reported paramyxovirus nucleocapsid assemblies in vitro.

Species	Ring-Like NC	Helical NC	Double-Headed NC
Parainfluenza virus 5 (PIV5)	+ ^1^	+	– ^2^
Measles virus (MeV)	+	+	–
Cetacean morbillivirus (CeMV)	–	+	–
Nipah virus (NiV)	–	+	+
Newcastle disease virus (NDV)	–	+	+
Sendai virus (SeV)	–	+	+
Mumps virus (MuV)	+	+	–

^1^ +: the assembly was reported; ^2^ –: no observed assembly yet.
